# Dataset on cigarette smokers in six South African townships

**DOI:** 10.1016/j.dib.2020.106260

**Published:** 2020-09-02

**Authors:** Nicole Vellios, Kirsten van der Zee

**Affiliations:** Research Unit of the Economics of Excisable Products, School of Economics, University of Cape Town, Cape Town, South Africa

**Keywords:** Smokers, Tobacco, Cigarettes, South Africa, Low socio-economic status, Township, Price, Illicit cigarettes

## Abstract

A total of 2453 smokers were interviewed in townships over two rounds of data collection. Townships are low-income, urban areas characterised by overpopulation, poor service delivery, crime, and poor socioeconomic outcomes. Township residents typically live in poverty. Data were collected from six townships in four of South Africa's nine provinces, namely Gauteng (Eldorado Park and Ivory Park), Western Cape (Khayelitsha and Mitchell's Plain), Free State (Thabong) and KwaZulu-Natal (Umlazi). These townships were chosen to represent both the geographical and racial spread of low socioeconomic areas in South Africa. Round 1 data (*n* = 1260) were collected from October to November 2017, and round 2 data (*n* = 1193) were collected from July to August 2018. The sample includes two of South Africa's four population groups: African and mixed race (locally referred to as “Coloured”, which describes people of mixed Khoisan, Malay, European, and black African ancestry). Since few Whites and Asians live in townships, they were not sampled. Households were selected via a random walk through each township. One smoker per household was interviewed (if a household contained at least one available smoker). We aimed to interview 200 adult smokers (aged 18+ years) per township per round. If a household had more than one smoker, a random selection determined which smoker to interview. Respondents were asked about their most recent cigarette purchase, specifically packaging type (single stick, pack, or carton), number of items purchased, brand, type of outlet where the cigarettes were bought, and the total amount paid for cigarettes. Respondents were also asked about other tobacco use in the household, and about their perceptions regarding illegal cigarettes. Socioeconomic and demographic information was collected at the individual and household level. The data has been used to estimate illicit trade (https://tobaccocontrol.bmj.com/content/early/2020/03/10/tobaccocontrol-2019–055136.info), and to analyse the determinants of smoking intensity (https://www.sciencedirect.com/science/article/pii/S2211335520300590).

## Specifications Table

**Table:** 

Subject	Public Health and Health Policy
Specific subject area	Tobacco control
Type of data	Excel file
How data were acquired	Questionnaires completed by interviewers using electronic devices
Data format	Raw
Parameters for data collection	Data were collected from low socio-economic areas in South Africa. The sample consisted of households with at least one cigarette smoker. If a selected household had more than one smoker, a random selection determined which smoker to interview.
Description of data collection	Random walk.
Data source location	The data were collected from six townships in four of South Africa's nine provinces: Gauteng (Eldorado Park and Ivory Park), Western Cape (Khayelitsha and Mitchell's Plain), Free State (Thabong) and KwaZulu-Natal (Umlazi). These townships were chosen to represent both the geographical and racial spread of low socioeconomic areas in South Africa.
Data accessibility	The data is available on a public repository. Repository name: DataFirst Direct URL to data: https://doi.org/10.25828/47cd-z315 The data is freely available after creating a user profile on the DataFirst website.
Related research article	Van der Zee, K, Vellios, N, Van Walbeek, C and Ross, H. 2020. The illicit cigarette market in six South African townships. Tobacco Control. doi: 10.1136/tobaccocontrol-2019–055,136 Boachie, M and Ross, H. 2020. Determinants of smoking intensity in South Africa: Evidence from township communities. Preventive Medicine Reports. doi: 10.1016/j.pmedr.2020.101099

## Value of the Data

• This dataset provides detailed information about tobacco use in South African townships. Smokers were asked about their smoking behaviour including: initiation age, number of cigarettes smoked per day, purchasing behaviour, brand choice, where they bought cigarettes, and quit attempts. Smokers are also asked about their use of other tobacco products, and perceptions regarding illicit cigarettes. The dataset provides detailed information on township smokers’ demographic and socio-economic characteristics (including gender, population group, age, and education).
• Researchers and policymakers who are interested in tobacco use in townships will benefit from this data.
• Researchers can investigate popular brands by township and province, as well as price differentials across brands/producers. Researchers can also compare prices charged by various types of retailers (foreign-owned spaza shops, South African-owned spaza shops, large retail stores, street stands, vending machine, house shop, internet, and from family/friends).
• Researchers can compare township smokers to the national smoking population (which can be obtained from national surveys). For example, one can compare smoking initiation age, smoking intensity and price paid between township smokers and smokers nationally. Further disaggregation can be done by race (mixed versus African), gender, age, employment status, education, province, and packaging type (single, packs or cartons).
Researchers can investigate smokers’ knowledge about what constitutes an illegal cigarette (tax not paid, low price, poor quality, no health waring health warning on pack, health warnings not written in English). Researchers can also investigate whether people are aware of the sale of illegal cigarettes in their township, ease of buying illegal cigarettes, where to buy illegal cigarettes from, and whether people would still buy cigarettes if they knew they the cigarettes were illegal.
Researchers can use the dataset to investigate the effectiveness of current health warnings on cigarette packs (written health warning). Smokers were asked if warning labels on cigarette packs led them to think about quitting in the past 30 days. In round 2, respondents were asked whether they had changed their smoking behaviour in the last seven to eight months. Researchers can investigate the reasons that people changed their behaviour. This can be useful from a policy perspective. For example, if people are concerned about health issues caused by smoking, education campaigns about the health effects may be useful in reducing smoking in low socio-economic areas.

## 1 Data Description

The data consists of two repeated cross sections. The first round of data (*n* = 1260) was collected from October−November 2017. A similar sized sample (*n* = 1193) was collected the following year from July−August 2018. Approximately 200 smokers per township were interviewed in each round (Table 1). In round 2, the goal of 200 smokers per township was not achieved in Eldorado Park (Gauteng) because of safety concerns. To compensate for the decreased number in Eldorado Park (*n* = 133), more smokers were interviewed in Ivory park (*n* = 263) (also in Gauteng). Table 1 below provides a description of the raw data for each round and overall.

**Table 1:** Description of data.

		Round 1	Round 2	Overall
**Total observations:**		1260	1193	2453
**Average:**				
Age		33.2	36.8	35.0
Daily cigarette consumption		9.6	9.1	9.4
**Proportion:**				
Male		69.4	73.1	71.2
Daily smokers		94.2	91.6	93.0
Female household head		34.8	39.0	36.7
Education	No formal schooling	2.2	1.3	1.8
	Some primary school completed	2.8	6.0	4.3
	Primary school completed	6.8	5.0	5.9
	Some secondary school completed	42.1	47.8	44.9
	Secondary school completed	41.9	33.3	37.7
	College/University completed	4.0	5.0	4.5
	Post graduate degree completed	0.2	1.5	0.9
Population group	African	65.6	72.2	68.8
	Mixed Race	34.4	27.7	31.1
	White	0.0	0.2	0.1
Employment Status	Employed	35.4	37.1	36.3
	Unemployed	44.0	46.1	45.1
	Not Economically Active	20.1	16.6	18.4
	Grant Holder	0.5	0.1	0.3
Township	Eldorado Park	17.1	11.2	14.2
	Ivory Park	16.4	22.1	19.2
	Khayelitsha	18.7	15.4	17.1
	Mitchell's Plain	18.3	16.7	17.5
	Thabong	16.0	17.0	16.5
	Umlazi	13.5	17.7	15.5

## 2 Experimental design, materials, and methods

### 2.1 Background and other similar surveys

Dedicated surveys that look at smoking behaviour have been done in many countries to monitor the tobacco epidemic. The Global Adult Tobacco Survey (GATS) is a nationally representative survey that has been conducted in more than 25 low- and middle-income countries. GATS enables countries to monitor adult tobacco use and assess key tobacco control measures, and is comparable across countries. [1] The Global Youth Tobacco Survey (GYTS), launched in 1999 is a school-based survey which monitors tobacco use amongst students aged 13−15. The GYTS was conducted in South Africa in 1999, 2002, and 2011. [2, 3] The International Tobacco Control Evaluation Project (ITC) is a system for evaluating the impact of tobacco control measures, particularly national policies of the World Health Organization's Framework Convention on Tobacco Control. ITC has been conducted in 29 countries and is also designed to be comparable across countries. [4] To date, neither GATS nor ITC have been conducted in South Africa.

Besides tobacco-specific surveys, national South African household surveys provide insight into tobacco use. South Africa has several nationally representative datasets that include questions on smoking: the National Income Dynamics Study, the Demographic and Health Survey, and the South African Health and Nutrition Examination Survey. [5], [6], [7] However, these surveys cover a range of topics; the focus is not on tobacco use and therefore questions on tobacco use are few. Although these surveys are nationally representative, they cannot be used to analyse tobacco use at a smaller geographical level, such as townships. For this reason, it was necessary to run a dedicated survey.

### 2.2 Survey design, sample selection, and data collection

South Africa has nine provinces. Data were collected from four provinces (Gauteng, Western Cape, KwaZulu-Natal, and the Free State). Since the Western Cape and Gauteng are highly populated, we selected two townships (one predominantly mixed race, the other predominantly African) in each of these provinces.

Interviewers walked through the selected townships and approached households. Questionnaires were completed using survey software (SurveyCTO) on a handheld device. Enumerators were required to enter the number of directions it was possible to take at an intersection, so that the system could randomly select the direction to take, the side of the road to walk along and the house to interview. The counting process occurred from the individual's left side. Due to safety issues, some round 1 fieldworkers deviated from this approach and went to areas that were perceived as more safe.

Fieldworkers compiled a roster of all adults (18+ years old) living in the household. The device randomly selected one smoker in the household to participate in the survey. Respondents had to be 18 years or older to be selected. If the selected smoker was unavailable to participate at the time of the visit, a second smoker was randomly selected.

This random walk methodology, while not rigorous, is the most affordable sampling method.

**Table 2:** List of variables.

Variable name	Variable description
round	Indicates either round 1 or round 2
hhgender	Gender of household head
hhsmoker_r2	Household head smoker (round 2 only)
hhage_r2	Household head age (round 2 only)
hhtobacco	Does anyone in this HH use other tobacco products?
hhtobtype	Which types of tobacco are used in the HH
hh_snuff	Snuff used in household
hh_chewing_tobacco	Chewing tobacco used in household
hh_ryo	Roll your own used in household
hh_ecig	E-cigarettes used in household
hh_wpipe	Waterpipe used in household
hh_cigars	Cigars used in household
hh_pipe	Pipe tobacco used in household
hhsize	Number of people in the household
hhadults	Number of adults in the household (18 and over)
gender	gender
race	population group
nationality	nationality
nationalityother	nationality other
age	age
educ	highest level of education completed
employ	Employment status
employother	employment status other
paid	Payment interval
use_cigarettes_r1	Respondent uses cigarettes daily or less than daily (round 1 only)
use_cigarettes_r2	Respondent uses cigarettes (round 2 only)
use_rolled_r2	Respondent uses roll your own (round 2 only)
use_cigars_r2	Respondent uses cigars (round 2 only)
use_pipes_r2	Respondent uses pipe tobacco (round 2 only)
use_snuff_r2	Respondent uses snuff (round 2 only)
use_chew_r2	Respondent uses chewing tobacco (round 2 only)
use_ecigarettes_r2	Respondent uses e-cigarettes (round 2 only)
use_water_r2	Respondent uses waterpipe (round 2 only)
freq_daily_cigarettes	Daily cigarette consumption (daily smokers only)
freq_daily_rolled	Daily use of roll your own by respondent
freq_daily_cigars	Daily use of cigars by respondent
freq_daily_pipes	Daily use of pipe tobacco by respondent
freq_daily_snuff	Daily use of snuff by respondent
freq_daily_chew	Daily use of chewing tobacco by respondent
freq_daily_ecigarettes	Daily use of e-cigarettes by respondent
freq_daily_water	Daily use of waterpipe by respondent
freq_weekly_cigarettes	Weekly cigarette consumption (less than daily smokers only)
freq_weekly_rolled	Weekly use of roll your own by respondent
freq_weekly_cigars	Weekly use of cigars by respondent
freq_weekly_pipes	Weekly use of pipe tobacco by respondent
freq_weekly_snuff	Weekly use of snuff by respondent
freq_weekly_chew	Weekly use of chewing tobacco by respondent
freq_weekly_ecigarettes	Weekly use of e-cigarettes by respondent
freq_weekly_water	Weekly use of waterpipe by respondent
start_age_r2	Age started smoking (round 2 only)
start_age_daily	Age started smoking daily
start_years_r2	How many years ago did you first start smoking cigarettes?
start_years_daily	How many years ago did you first start smoking cigarettes daily?
start_age_lessdaily_r1	Less than daily smokers only: Age started smoking (round 1 only)
start_years_lessdaily_r1	Less than daily smokers only: how many years ago start smoking (round 1 only)
cigcons_wkly_pd	Weekly cig consumption converted to daily (derived)
cigcons	Daily cig consumption, including daily and weekly smokers (derived)
packtype	Recent purchase: packaging types bought
packtype_cart	Recent purchase: bought carton
packtype_pack	Recent purchase: bought pack
packtype_stick	Recent purchase: bought single stick
cart_numitem	Recent purchase: number of items (cartons)
cart_numpcks	Recent purchase: number of packs (per carton)
cart_cigppck	Recent purchase: number of cigarettes per pack (cartons)
numsticks_cart	Recent purchase: derived, total number of cigarettes (cartons)
cart_exp	Recent purchase: total expenditure (cartons)
cart_priceeach_r2	Recent purchase: reported price per carton (round 2 only)
cart_brand	Recent purchase: brand (cartons)
cart_brandother	Recent purchase: brand, other (cartons)
price_cart	Recent purchase: derived price per stick (cart_exp/numsticks_cart)
pck_numitem	Recent purchase: number of items (packs)
pck_cigppck	Recent purchase: number of cigarettes per pack (packs)
numsticks_pck	Recent purchase: derived, total number of cigarettes (packs)
pck_exp	Recent purchase: total expenditure (packs)
pck_priceeach_r2	Recent purchase: reported price per pack (round 2 only)
pck_brand	Recent purchase: brand (packs)
pck_brandother	Recent purchase: brand, other (packs)
price_pck	Recent purchase: derived price per stick (pck_exp/numsticks_pck)
stick_numitem	Recent purchase: number of items (single sticks)
stick_exp	Recent purchase: total expenditure (single sticks)
stick_priceeach_r2	Recent purchase: reported price per single stick (round 2 only)
stick_brand	Recent purchase: brand (single sticks)
stick_brandother	Recent purchase: brand, other (single sticks)
price_stick	Recent purchase: derived price per stick (stick_exp/stick_numitem)
shop	Recent purchase: store type
shopother	Recent purchase: store type, other
shop_reas	Recent purchase: reason for choosing store type
shop_reas1	Recent purchase: store close to home
shop_reas2	Recent purchase: work close to store
shop_reas3	Recent purchase: store is cheap
shop_reas4	Recent purchase: bought other things at store
shop_reas5	Recent purchase: like this store
shop_reasother	Recent purchase: reason selected store, other
share	Share cigarettes with your household members
illegal_untaxed	Believe cig illegal if: tax unpaid
illegal_lowprice_r1	Believe cig illegal if: price low (round 1 only)
illegal_lessR1_r2	Believe cig illegal if: price R1/cig or less (round 2 only)
illegal_lowquality	Believe cig illegal if: low quality
illegal_healthwarning	Believe cig illegal if: no health warning
illegal_warningnoteng	Believe cig illegal if: health warning another language
illegal_none_r2	Believe cig illegal if: none (round 2 only)
knowtax_r2	Would people buy cigs if they knew the tax had not been paid (round 2 only)
illegal_aware	Do you think illegal/smuggled cigarettes are available for sale in your area
illegal_easybuy	Is it easy to buy illegal cigarettes in your area
where	Where can people find illegal cigarettes?
shop_foreign	Where can you buy illegal cigs: foreign owned spaza
shop_local	Where can you buy illegal cigs: SA owned spaza
shop_dk_owner	Where can you buy illegal cigs: spaza, ownership unknown
shop_retail	Where can you buy illegal cigs: retail store
shop_stand	Where can you buy illegal cigs: street stand
shop_vend	Where can you buy illegal cigs: vending machine
shop_house	Where can you buy illegal cigs: house shop
shop_online	Where can you buy illegal cigs: internet
shop_ff	Where can you buy illegal cigs: family/friends
shop_other	Where can you buy illegal cigs: other
shop_defother	Where can you buy illegal cigs: other, specify
wouldbuyillegal_r1	If people know certain cigs are illegal, would they still buy (round 1 only)
tryquit	Tried quitting in past 12 months
warningquit	In the past 30 days, have warning labels led you to consider quitting
language	interview language
reliable	interview reliable
police	Respondent concerned fieldworker affiliated with police
comment	interviewer feedback
township	Township
dwelling	Dwelling type
hhelectricity	electricity
hhtoilet	flush toilet
hhsatellite	satellite
hhcellphone	cell phone
hhtelevision	television
hhradio	radio
hhfridge	fridge
hhcar	car
hhmotorbike	scooter/motorcyle
hhwashingmach	washing machine
hhmicrowave	microwave
hhstove	stove
province	Province
date	Date of interview
compare_smoke_r2	At the beginning of this year (Jan), did you smoke cigarettes? (round 2 only)
compare_brands_r2	In January, what brand of cigarettes did you smoke most often? (round 2 only)
compare_freq_r2	In January, how many cigarettes PER DAY did you smoke on average? (round 2 only)
compare_purchase_r2	In Jan., did you usually buy cartons, packs or loose cigarettes? (round 2 only)
compare_change_r2	Changed your cigarette smoking behaviour between Jan. & now? (round 2 only)
compare_reason_r2	Reason for change in smoking behaviour between January and now (round 2 only)
compare_reason_1_r2	I am trying to reduce the health impact of smoking (round 2 only)
compare_reason_2_r2	Cigarettes are becoming more expensive (round 2 only)
compare_reason_3_r2	The quality of cigarettes decreased (round 2 only)
compare_reason_4_r2	The quality of cigarettes increased (round 2 only)
compare_reason_5_r2	I can afford more cigarettes (round 2 only)
compare_reason_6_r2	I enjoy smoking more cigarettes (round 2 only)
compare_reason_7_r2	Pressure from family or friends (round 2 only)
compare_reason_6666_r2	Reason for change: Other (round 2 only)
compare_reason_7777_r2	Reason for change: None (round 2 only)
compare_reason_9999_r2	Reason for change: Don't know (round 2 only)
compare_reason_8888_r2	Reason for change: Refuse (round 2 only)
compare_reasonother_r2	Reas. for change in smoking behaviour betw. January - now, other (round 2 only)

Note: Spaza shops are informal convenience shops.

### 2.3 Variable definition

There are 157 variables in the final dataset (Table 2). The variables for the two rounds are standardized, with the inclusion of a variable labelled “round”, which indicates when the data were collected (round 1 or round 2). Observations are appended to produce the final dataset. There were three variables that only appeared in round 1 and 35 variables that appeared only in round 2 (these variables are suffixed with “_r1” and “_r2”) . The difference in these variables across the rounds is due to minor edits to the questionnaire.

## Ethics statement

The University of Cape Town's Research in Ethics Committee (Faculty of Commerce) approved this research (REC2017/10/011). Informed consent was obtained from all participants.

## Declaration of Competing Interest

The authors declare that they have no known competing financial interests or personal relationships which have, or could be perceived to have, influenced the work reported in this article.

## Appendix Supplementary materials

Image, application 1

Image, application 2
